# The PRCI study: design of a randomized clinical trial to evaluate a coping intervention for medical waiting periods used by women undergoing a fertility treatment

**DOI:** 10.1186/1472-6874-13-35

**Published:** 2013-09-03

**Authors:** Henrietta DL Ockhuijsen, Agnes van den Hoogen, Nickolas S Macklon, Jacky Boivin

**Affiliations:** 1 Department of Reproductive Medicine and Gynaecology, University Medical Centre Utrecht, Utrecht, the Netherlands; 2 Department of Neonatology, Wilhelmina Children’s Hospital and University Medical Centre Utrecht, Utrecht, the Netherlands; 3 Department of Obstetrics and Gynaecology, Academic Unit of Human Development and Health, University of Southampton, Southampton, UK; 4 School of Psychology, Cardiff University, Cardiff, UK

**Keywords:** Coping-intervention, Anxiety, Medical waiting period, Fertility, Randomized clinical trial

## Abstract

**Background:**

Many medical situations necessitate a stressful period of waiting for potentially threatening test results. The medical waiting period is often associated with negative anticipatory anxiety and rumination about the outcome of treatment. Few evidence-based self-help coping interventions are available to assist individuals manage these periods. Theory and research suggest that positive reappraisal coping strategies may be particularly useful for this type of unpredictable and uncontrollable stressful context. The objective of this study is to investigate the effects of a Positive Reappraisal Coping Intervention (PRCI) on psychological well-being of women waiting for the outcome of their fertility treatment cycle.

**Methods/Design:**

In a three-armed randomized controlled trial, the effectiveness of the PRCI will be tested. Consecutive patients undergoing in vitro fertilisation in a Dutch university hospital and meeting selection criteria will be invited to participate. Those who agree will be randomized to one of three experimental groups (N=372). The PRCI Intervention group will receive the intervention that comprises an explanatory leaflet and the 10 statements designed to promote positive reappraisal coping, to be read at least once in the morning, once in the evening. To capture the general impact of PRCI on psychological wellbeing patients will complete questionnaires before the waiting period (pre-intervention), on day ten of the 14-day waiting period (intervention) and six weeks after the start of the waiting period (post-intervention). To capture the specific effects of the PRCI during the waiting period, patients will also be asked to monitor daily their emotions and reactions during the 14-day waiting period. The primary outcome is general anxiety, measured by the Hospital Anxiety and Depression Scale. Secondary outcomes are positive and negative emotions during the waiting period, depression, quality of life, coping and treatment outcome. During recruitment for the RCT it was decided to add a fourth non-randomized group, a PRCI Control group that received the PRCI and completed the questionnaires but did not complete daily monitoring.

**Discussion:**

Positive reappraisal is one of the few ways of coping that has been shown to be associated with increased wellbeing during unpredictable and uncontrollable situations like medical waiting periods. A simple evidence based self-help intervention could facilitate coping during this common medical situation. This RCT study will evaluate the value of a self-help coping intervention designed for medical waiting periods in women undergoing fertility treatment.

**Trial registration:**

The study is registered at the Clinical Tials.gov (NCT01701011).

## Background

The diagnosis and treatment of various medical conditions requires patients to wait for results that are potentially threatening to their well-being (e.g., breast biopsy results, pregnancy test results after fertility treatment, genetic screening outcomes) [1]. The outcomes of these tests are often unpredictable and often cause high levels of anticipatory anxiety and uncertainty [2] that are difficult to cope with. As the outcome of the medical tests or procedures for which patients are waiting cannot be changed or controlled, there is little point for the patient in trying to alter the situation. Instead, coping efforts should be directed at regulating the negative anticipatory emotions associated with waiting (e.g., feeling nervous, tense, worried, anxious) [3]. Despite the pervasiveness of medical waiting periods, few studies have investigated coping interventions to manage this stressful medical context [4].

Meaning-based coping strategies have been observed to be effective in contexts that involve a sustained period of unpredictability and uncertainty. Tedlie Moskowitz et al. [5] and Folkman and Moskowitz [6] observed that even in very stressful and uncertain situation such as caring for a terminally ill partner participants reported experiencing positive feelings. One type of coping strategy associated with these positive psychological states was positive reappraisal coping, which helped the person to redefine the situation in a more positive way, allowing them to derive some benefit from the negative experience. Folkman and Lazarus [3] proposed that these positive emotions had an important role in motivating people to continue in their efforts to cope in these ongoing stressful situations.

Fertility treatment is an example of a medical context that requires patients to wait for several weeks for the outcome of their treatment. The use of Assisted Reproductive Techniques (ART) (e.g., In Vitro Fertilization (IVF) and IntraCytoplasmic Sperm Injection (ICSI)) continues to increase and worldwide the total number of babies born through IVF is now exceeds 5 million [7].

A cycle of in vitro fertilisation typically requires nine to 12 days of self-injection with potent fertility drugs to stimulate the production of oocytes (eggs), retrieval of oocytes via trans-vaginal ultrasonography, fertilisation of oocytes in the laboratory with partner or donor sperm, and transfer of the resulting embryo to the uterus. Couples then wait two weeks to find out whether implantation and a pregnancy have occurred. Women often report that IVF/ICSI is an emotional and physical burden that can cause anxiety and stress [8,9]. The aspects most frequently reported as stressful are the fourteen-day waiting period between embryo transfer and the pregnancy test, and being informed that the treatment was unsuccessful [8-12]. Symptoms of anxiety and depression have been shown to increase during the waiting period after ET [1,2,10,11]. The lack of control the patient has to influence outcome during this period has also been shown to contribute to the increase in distress [2].

Despite the emotional stress reported during fertility treatment, many women do not seek professional support [13,14]. The stated reasons for this are often practical, such as the costs of counselling, distance to the appointment or being unsure how to arrange an appointment [13]. Nevertheless, women still worry about the impact of stress on the outcome of treatment and available meta-analyses are inconclusive on such effects [15,16]. A meta-analysis of 31 prospective studies found a small but significant association between stress, distress and reduced pregnancy chances [15]. Another meta-analysis of 14 prospective studies found no significant effect of emotional distress on the chance of becoming pregnant [16]. Several psychosocial interventions designed to support women with infertility have been described [17,18]. However, these are mostly aimed at providing general support throughout the entire fertility treatment and not at a specific point in the treatment, such as the waiting period. The impact of psychosocial interventions on anxiety, depression, coping and treatment outcome is inconsistent across reviews. One review of 25 studies concluded that psychosocial interventions reduced negative affect such as anxiety and infertility-specific distress but had no effect on pregnancy rates [17]. A subsequent meta-analysis of 22 studies concluded that psychotherapy was effective in reducing anxiety delivered in individual and group format [19]. The pregnancy rate with assisted reproductive techniques (ART) was similar in individual and group therapy however there was a difference in pregnancy rates between psychotherapy (45%) and the control group (14%) [19]. A final meta-analysis of 21 controlled studies showed that psychosocial interventions did not impact any form of psychological distress but the meta-analysis for pregnancy rate was significant with sub-group analysis showing increased pregnancy rate but only in patients undergoing non-ART treatments [18]. Inconsistent results could be due to interventions being too general. Indeed, Boivin [17] found that interventions with a strong educational and skills component that focused on specific targets (e.g., coping training, sex during fertile period) were more effective than those focused primarily on emotional expression and support.

The Positive Reappraisal Coping Intervention (PRCI) was designed to address unmet coping needs during medical waiting periods such as waiting for fertility treatment results. During the stressful waiting period after ET, women do not normally attend a clinic for tests or procedures and therefore lose the opportunities they had to receive informal support from the medical staff, clinic or other patients undergoing treatment at the same time [1]. Individual counselling of women during the waiting period is often not possible for hospitals because of the time investment and costs [13]. The PRCI is self-administered and comprises an explanatory leaflet describing this method of coping and ten statements designed to promote positive reappraisal coping. PRCI was conceptualised from the cognitive model of stress and coping [20,21] and developed according to the Medical Research Council framework for developing complex interventions [1]. Lancastle and Boivin [1] investigated the acceptability and feasibility of the intervention in an RCT of 55 women who used PRCI during the waiting period of an IVF/ICSI cycle versus a control group reading ten control positive statements. Results showed that the PRCI group rated the intervention as more helpful and suitable for the IVF/ICSI situation, more able to help women feel positive as well as better in sustaining positive reappraisal coping strategies during the waiting period. A feasibility study in the Netherlands showed that it is possible to recruit the amount of women necessary for the RCT in two years. Furthermore, the majority of the 27 women included in the study found the PRCI was suitable and feasible. (unpublished data, Ockhuijsen). These results suggest that PRCI could be useful for medical waiting periods. However, its effectiveness on general or treatment-specific anxiety has not yet been systematically evaluated in a randomized controlled trial.

## Methods/Design

The PRCI will be evaluated in a three-arm Randomized Controlled Trial (RCT). Participants will be randomized to an intervention, monitoring control group or routine care. To capture the general impact of the PRCI all three groups will complete questionnaires at three time points: just before the waiting period (Time 1: pre-intervention), on Day 10 of the 14-day waiting period (Time 2: intervention) and 6 weeks after the start of the waiting period (Time 3: post-intervention). Table 1. Mobile phone text reminders will be sent to patients about completing the Time 1 and Time 3 questionnaires (if necessary) and all patients will receive a reminder just prior to the Time 2 assessment on the ninth day of the waiting period.

**Table 1:** Measurement timetable according to group

**Measurements**	**T1 hCG to Embryo transfer**	**T2 Day 10 waiting period**	**T3 Six weeks after ET**
**BIF**	1,2,3,4	------	------
**HADS**	1,2,3,4	1,2,3,4	1,2,3,4
**FertiQol**	1,2,3,4	1,2,3,4 (Treatment only)	1,2,3,4 (non-pregnant group only)
**WCQ**	1,2,3,4	1,2,3,4	1,2,3,4
**DRK one day**	1,2,3,4	3,4	------
**IEF**	------	1,4	------
**DRK daily**	------	1,2	------
**Medical chart review**	------	------	1,2,3,4

Note. Numbers refer to group: 1 = PRCI Intervention, 2 = Monitoring Control, 3 = Routine Care and 4 = PRCI Control (non-randomized). BIF = Background Information Form, HADS = Hamilton Anxiety and Depression Scale, FertiQol = The Fertility Quality of Life, WCQ = Ways of Coping Questionnaire, DRK = Daily Record Keeping, IEF = Intervention evaluation form.

To capture the specific impacts of PRCI on the waiting period the intervention group will rate daily their emotions and reactions during the 14-day waiting period. Daily monitoring has previously been shown to be an efficient and sensitive way of evaluating emotional reactions during fertility treatment [2,9] and to be sensitive to intervention effects during ART [22]. One potential drawback of this method of assessment is that it may impact on the reporting of emotions itself. For example, habituation or sensitisation to monitoring per se may decrease or increase reporting of anxiety compared to groups that do not monitor [23]. Due to this potential reactivity the monitoring control group will also monitor emotions and reactions daily during the waiting period. The routine care control group will not receive the intervention or monitor reactions.

During recruitment for the RCT it was decided to add a fourth non-randomized group. The decision to add this group was based on preliminary results of on-going qualitative research with the PRCI and daily monitoring among women who had experienced miscarriages (unpublished data, Ockhuijsen). The women in the miscarriage study reported that the daily monitoring had an effect on their emotions in that it helped them take account of the impact of miscarriages on their emotional lives. If daily monitoring was itself an intervention then it could attenuate, heightened or obscure effects of the PRCI intervention in unknown ways. Therefore it was decided to add a PRCI Control group that received the PRCI and completed the questionnaires but did not complete daily monitoring.

### Participants/recruitment

**Figure 1:**
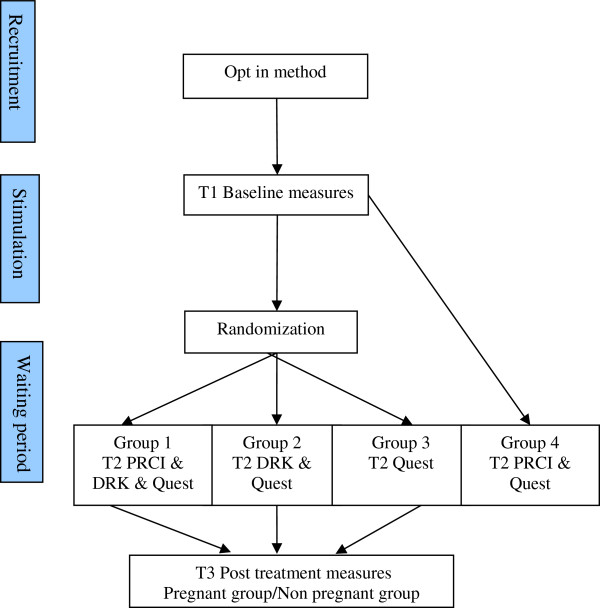
Flow chart study protocol.

The RCT will be conducted over a two-year period in a fertility clinic at a University hospital in the Netherlands. See Figure 1 for the flow chart of the study. The opt-in method will be used to recruit participants. In this method participants are sent an invitation to the trial and themselves contact the team to take part. The inclusion criteria will be all women undergoing a stimulated or cryopreserved IVF/ISCI treatment. The exclusion criteria will be women not speaking the Dutch language. All women meeting these criteria and starting a treatment will be sent a letter with general information about the study. Doctors and nurses working in the fertility clinic will remind women of the letter and the study. Patients who are interested in the study will return a reply form or e-mail indicating their interest and a researcher will contact them to give more information about the study and answer any questions. Those who decide to participate will be sent a written information sheet and a consent form to return in a pre-addressed stamped envelope. During their first visit to the hospital, more information will be given about the logistics of the study, as needed, but all patients will be given the same information according to a written protocol.

The fourth group will be recruited as for the RCT. The fourth group will receive the same information as the participants from the RCT. Although this group will not be randomized, participants will be told that randomization will take place. Participants will be fully debriefed about the need for this deception at the end of the study.

The Ethical committee of the University of Utrecht provided ethical review and approval for this study, including the addition of the fourth non-randomized group (protocol number 10-174/K).

### Sample size

The sample size calculation for the three-arm RCT is based on the following parameters. To test the difference on anxiety between three groups using a mixed factorial ANOVA, power of 95%, α = 0.05 and a medium effect size a total of 297 subjects was required (99 patients per group) [24,25]. However, taking into account a 20% attrition rate at least 124 women will be recruited in each group. Effect size and attrition were derived from Lancastle and Boivin [1]. The addition of the fourth group only slightly modified sample size and 110 participants were recruited to the fourth group.

### Randomization

Stratified randomization of the 372 women into one of the three groups will be performed by using a computer-generated table of random numbers. The type of treatment (stimulated or cryopreserved IVF/ICSI) will stratify the population because emotions and expectations relative to a stimulated IVF/ICSI may differ from a cryo-preserved treatment [26,27]. Randomization will take place after the first assessment (Time 1 pre-intervention) between follicle aspiration and embryo transfer. An independent research nurse will be responsible for the randomization. Double blinding will take place for participants and clinic staff. Participants will not be told what intervention is being evaluated. The researcher will have no contact with participants after randomization. All women will receive written information about group assignment on the day of the embryo transfer. They will receive instructions for the waiting period in an opaque sealed envelope after the embryo transfer. The clinical staff that performs the embryo transfer will be blinded for the content of the envelope. After the embryo transfer, there will be no further contact between the clinical staff, other patients, or the researcher during the 14-day waiting period.

### Intervention and control groups

Patients will be randomly assigned to one of the three groups: PRCI Intervention, Routine Care Control and Monitoring Control. In addition data will be collected for the PRCI Control.

**Figure 2:**
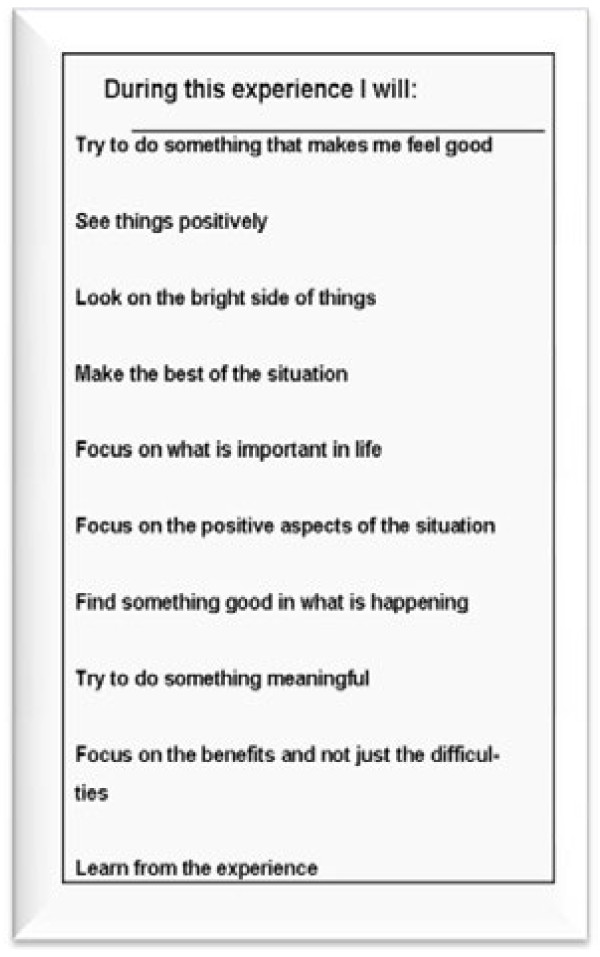
**PRCI intervention.** © 2008 by Cardiff University. All rights reserved. No part of this document may be reproduced or transmitted in any form or by any means, electronic, mechanical, photocopying, recording, or otherwise, without prior written permission of authors.

The PRCI Intervention and PRCI Control groups will receive the PRCI. The PRCI is a small card that contains ten positive reappraisal statements and a leaflet with a detailed explanation about this coping approach. See Figure 2 for the PRCI card. Women will be instructed to read the PRCI at least twice a day, once in the morning and once in the evening as well as at any time they feel the need, and to think about how each statement applies to them personally. The other groups will not receive the PRCI. The Monitoring Control group will complete daily monitoring and questionnaires, whereas the Routine Care Control and PRCI Control groups will only complete questionnaires.

### Objective

The aim of this study is to investigate the effect of the PRCI on psychological well-being of women waiting for the results of an IVF/ICST treatment. The primary outcome is level of general anxiety measured before the waiting period (pre-intervention), on day ten of the 14-day waiting period (intervention) and six weeks after the start of the waiting period (post-intervention). Secondary outcomes are positive and negative treatment emotions during the waiting period, depression, coping style, quality of life and treatment outcome.

### Hypotheses

The PRCI increases the use of positive reappraisal coping strategies and reduces symptoms of general anxiety and depression, improves quality of life across assessment (pre intervention, intervention and post intervention) as well as increases positive and decreases negative emotions during the fourteen days of the waiting period in patients undergoing an IVF/ICSI treatment.

### Study measures

Data will be obtained with self-reported questionnaires, daily monitoring and from the treatment medical records. Table 1 shows the assessment timetable. Questionnaires will be completed prior to, during and after the intervention whereas monitoring will be daily during the two-week waiting period. The following self-report measures will be used:

The Background Information Form (BIF) is a 16-item questionnaire to measure demographic (e.g. age, educational status), medical (e.g. previous illness) and gynaecological (e.g. infertility diagnosis, previous infertility treatment) characteristics as well as treatment expectations (e.g. chances of conceiving, perceived control over the outcome).

The Hospital Anxiety and Depression Scale (HADS) will be used to measure general anxiety and depression. The HADS consists of 14 items (7 items for each subscale) that are rated on a 4-point Likert scale. The total score is the sum of the 14 items, and for each subscale the score is the sum of the respective seven items (ranging from 0–21). Scores on each scale can be interpreted in ranges: normal (0–7), mild (8–10), moderate (11–14) and severe (15–21). The Dutch version of the HADS has been shown to be a valid and reliable instrument [28].

The Fertility Quality of Life (FertiQoL) scale will be used to measure the impact of infertility and its treatment. FertiQoL consist of 36 items that assess core (24 items) and treatment related quality of life (10 items) and overall life and physical health (2 items). Items are rated on a 5-point response scale. Cronbach reliability coefficients for the Core and Treatment FertiQoL were 0.92 and 0.81, respectively [29]. The convergent validity of the Dutch version of the FertiQoL has been investigated and shows similar reliability [30]. Items are summed and scaled, with a range of 0 to 100. Higher scores indicate better quality of life.

The Ways of Coping Questionnaire (WCQ) will be used to measure use of coping strategies. The WCQ is based on the cognitive stress and coping model of Lazarus and Folkman [31,32]. The instrument is designed to measure situation-specific coping. The 41 items of the Dutch version are rated on a 4-point response scale that, when summed, yields 6 subscales. Taking responsibility (6 items), problem solving (8 items), social support (6 items), wishful thinking (8 items), avoidance (7 items), positive reappraisal (6 items). The Cronbach alpha coefficients reported for the six subscales ranges from 0.65 to 0.80. To measure the concurrent validity the WCQ has been compared with the seven scales of the Utrecht Coping List with good correspondence between these [33].

The Intervention evaluation form (IEF) is a 24-item questionnaire developed to assess intervention feasibility, acceptability and effects, which was used to assess PRCI in previous research [1]. It measures the following aspects of the intervention: practicality (6 items), acceptability (4 items), endorsement (4 items), perceived psychological effects (8 items) and perceived duration of intervention effects (2 Items).

The Daily Record Keeping (DRK) sheet will be used daily to rate reactions to the 14-day waiting period (PRCI Intervention and Monitoring Control groups only). The DRK comprises 46 possible reactions to the IVF waiting period, including 20 emotions, optimism and pessimism about pregnancy, 12 physical symptoms, five appraisals, and seven coping strategies. The emotional subscale is based on the theory of Lazarus and Folkman [20] and contains affective reactions that are averaged to produce anxiety (i.e., tense, nervous, worried), depression (i.e., anger, frustrated, sad) and positive affect (i.e., happy, content, fulfilled). The five coping strategies are measured with Stone and Neale’s [34] daily coping measure namely strategies of distraction, positive redefinition, problem-focused, seeking emotional support and acceptance. The twelve physical symptoms list two common side effects of medication (i.e., abdominal discomfort, spotting), two symptoms related to treatment success/failure (e.g., breast tenderness, menstrual cramps) and the remaining eight symptoms originate from the physical stress reactions (e.g., racing heart, muscle tension) of the Pennebaker Inventory of Limbic Languidness (PILL) [1]. Subscale scores were created (where relevant) by averaging across subscale items and higher scores indicate more of the attribute (e.g., more tension, breast tenderness, distraction). Participants will be instructed to complete the DRK at the end of the day, and for the PRCI intervention group at least one hour after reading the PRCI card to limit the chance of DRK ratings being artificially and transiently influenced by completing the DRK.

The DRK has been shown to be acceptable for monitoring during protracted periods of treatment with 15% attrition during 75 days of monitoring and 9% over a period of 30 days [9]. The reported Cronbach alpha ranges from 0.70 – 0.82 for subscales [2]. The DRK was translated and used in a Dutch study [22] that showed good correspondence between the original and Dutch version, and acceptable convergent and discriminant validity with other measures of anxiety and depression. The DRK was also tested in a feasibility study in a population from the recruitment clinic in the Netherlands showing acceptability and feasibility (unpublished data, Ockhuijsen).

A medical chart review at the end of treatment will be used to obtain treatment data and will include the total number of past IVF/ICSI stimulated and cryopreserved cycles, past intrauterine insemination cycles, previous conceptions/births, infertility diagnosis, smoking status, alcohol use, and body mass index. Data about the current treatment will include: type of treatment and protocol, date of first and subsequent treatment attempt, treatment cancellation (yes/no) and reason for cancellation, number of oocytes retrieved, total number of embryos created, transferred and cryopreserved, treatment outcome (positive pregnancy test, clinical pregnancy, live birth), physician recommendation for next cycle and patient compliance with physician recommendation”.

### Statistical analysis

IBM SPSS Statistics will be used to perform the statistical analysis. Equivalence of baseline measures between groups will be examined by one-way analyses of variance (ANOVA) for normally distributed variables on interval or ratio level, chi-square for normally distributed variables on nominal level and Kruskal Wallis *H* test for normally distributed variables on ordinal level. If study variables are not normally distributed then data will be transformed to normalise (e.g., square root, log, etc. as is required). If the groups are not comparable on demographics, medical history or gynaecological variables, those variables will be employed as covariates in subsequent analyses. To examine the differences between groups over time a repeated measure mixed factorial ANOVA design will be used for variables on interval or ratio level. To compare the fourth group with the three separate groups also an ANOVA design will be used. Intention to treat and multi-level modelling will be used to analyse the data to take account of attrition.

## Discussion

Waiting for a potentially threatening medical test result in a period where there is no control in the outcome is very stressful and can bring about feelings of tension, nervousness and worry. The Positive Reappraisal Coping Intervention (PRCI) investigated in this RCT was specifically designed to help people cope with these medical waiting periods. Positive reappraisal coping is a set of strategies in which the significance of the event is reinterpreted in a more positive way [6]. In the present study, we will evaluate the effectiveness of the PRCI on psychological wellbeing of woman waiting for the results of an IVF/ICSI treatment cycle. Although the effectiveness of the PRCI has not yet been investigated, the coping literature shows that positive reappraisal strategies are one of the few ways of coping that are associated with increased positive affect and sustained ability to cope in unpredictable and uncontrollable stressor situations [6]. The results of this study could therefore be important because there is a lack of inexpensive self-help evidence based coping-interventions that could be used during the common medical waiting period.

This study has several strengths. This RCT was developed based on the results of several previous studies. The framework for developing complex interventions was used to design the PRCI and provide evidence of feasibility and acceptability in the studied population [1,35]. The MRC framework guides development to be theory based to create active components that can be delivered effectively during the RCT. Also the development and validation of the assessment tools was based on previous research showing good psychometric properties in Dutch translations for the key measures [1,9]. We chose a three-armed RCT to ensure balance between the determination of the general impacts of PRCI measured by questionnaires and its more specific impacts on the waiting period measured by monitoring. The Monitoring Control group of women who only monitor will allow us to disentangle effects due to PRCI and those due to monitoring per se on emotions and reactions during the waiting period. The RCT procedures match those of high quality trials with randomization and allocation concealment, blinding of patients and clinical staff [36].

The decision to add the PRCI Control after the RCT has the disadvantage that this group will not be randomized. However, the group will allow us to estimate PRCI effects independent of daily monitoring, in case monitoring has an unexpected intervention effect that interacts with PRCI effects. Our RCT procedures use written protocols and blinding which should minimise the chance of systematic bias even in this non-randomized group with respect to attributes that may affect the dependent variables. The expected sample size will provide adequate power for detection of effects, determined from previous studies using the PRCI tool.

A limitation of this study is the method used for recruiting the participants. The opt-in method was employed to recruit on the advice of the Ethics Committee. In this method participants are sent an invitation to the trial and themselves contact the team to take part, which differs from the more conventional opt-out approach where all patients are contacted about the trial unless they have contacted the team to indicate that they do not wish to be approached. Although the opt-out method improves recruitment, the ethical committee often does not approve this method because repeated contact is too burdensome for participants [37,38]. In the study of Junghans at al. [37] there was a difference in risk factors between the two methods. Patients in the opt-in arm had fewer risk factors (44%) compared to patients in the opt-out arm (60%) (P = 0.053).

Another limitation could be the participation of only one hospital. We choose to recruit in only one hospital because this university hospital attracts patients from all over the country. However, as there is collaboration with other clinics nationwide inferences about the generalizability of findings can be determined via comparison of patient characteristics collected as part of wider collaborations.

## Abbreviations

ANOVA: Analyses of variance; ART: Assisted reproductive techniques; DRK: Daily record keeping; ET: Embryo-transfer; FertiQol: Fertility quality of life; HADS: Hospital anxiety and depression scale; IVF: In vitro fertilisation; IEF: Intervention evaluation form; ICSI: Intra cytoplasmic sperm injection; IUI: Intra uterine insemination; MRC: Medical research council; PMI: Positive mood intervention; PRCI: Positive reappraisal coping intervention; PIA: Pre intervention assessment; RCT: Randomized controlled trail; WCQ: Way of coping questionnaire.

## Competing interests

The authors declare to have no financial or non-financial conflicts of interest.

## Author’s contributions

HDLO, AH, NSM and JB contributed to the design of the study and identified the research questions and hypotheses. HDLO and AH were responsible for obtaining ethics approval. JB and HDLO planned the statistical analyses. HDLO is responsible for the study implementation. JB, AH and NSM are scientific reviewers for the project. All authors have read, revised, and approved the final manuscript.

## Pre-publication history

The pre-publication history for this paper can be accessed here:

http://www.biomedcentral.com/1472-6874/13/35/prepub
